# *Limosilactobacillus reuteri* normalizes gut microbiota dysfunction and social deficits of rat offspring associated with prenatal exposure to stress

**DOI:** 10.1080/19490976.2026.2649440

**Published:** 2026-03-30

**Authors:** Fushen Zhang, Weiye Xu, Ru Zeng, Jie Chen, Jufang Huang

**Affiliations:** a Department of Anatomy and Neurobiology, Xiangya School of Basic Medical Sciences, Central South University, Changsha, People's Republic of China; b Key Laboratory of Model Animals and Stem Cell Biology in Hunan Province, Hunan Normal University Health Science Center, Changsha, People's Republic of China

**Keywords:** Prenatal stress, *Limosilactobacillus reuteri*, social deficits, oxytocin

## Abstract

Prenatal stress (PS) is a potential risk factor for social behavior impairment in offspring. Here, we demonstrate that PS induces gut microbiota alterations that are associated with impaired sociability and social novelty preference in rat offspring. In addition, we found that these behavioral deficits could be partially rescued through either cohousing with normal offspring or fecal microbiota transplantation from control donors. Metagenomic analysis identified *Limosilactobacillus reuteri* (*L. reuteri*) as a key species based on the considerable difference in its abundance between the PS and control offspring. Subsequent investigations revealed that supplementing *L. reuteri* during critical neurodevelopmental windows restored oxytocin levels in the paraventricular nucleus (PVN) and rescued dopamine reward pathway function, thereby ameliorating PS-induced social deficits. Notably, these beneficial effects were completely abolished by either treatment with an oxytocin receptor antagonist or subdiaphragmatic vagotomy. Thus, both oxytocin signaling and vagal afferent pathways play essential roles in the observed benefits of *L. reuteri*. Our findings indicate that social behavior impairments in offspring exposed to prenatal maternal stress can be explained by a novel mechanism involving the gut microbiota–brain axis: whereby PS-induced depletion of specific commensal bacteria (particularly *L. reuteri*) disrupts vagus nerve-mediated oxytocinergic modulation of PVN-to-VTA dopaminergic circuits, ultimately leading to social behavior impairments in offspring.

## Graphical abstract

**Figure:**
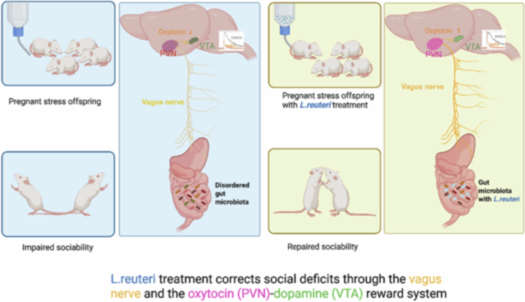

## 1. Introduction

Research on prenatal stress and maternal stressful life events has shown that they are closely related to children's neurodevelopment. Maternal stress during pregnancy increases the risk of social function deficits in offspring, among which autism spectrum disorder is a representative disease of social function deficits, with core features including social communication deficits and repetitive, stereotyped behaviors or interests. It affects an estimated 2.3% of children and around 2.2% of adults in the United States,^1^^,^^2^ with a significantly higher prevalence in males.^3^^,^^4^ Research focusing on prenatal stress^5-7^ and maternal stressful life events has demonstrated a substantial association with child development.^8^ Moreover, maternal emotional states during pregnancy have been linked to an increased risk of autism and the manifestation of autism-like traits in offspring.^9^^,^^10^ In addition, preclinical studies have shown that male offspring exposed to prenatal stress exhibit elevated mRNA levels of IL-1β and IL-6 in the cerebral cortex, reduced serotonin metabolism, impaired social interaction, and increased corticosterone levels following social interaction.^11^ Another study suggested that prenatal stress exposure may predispose offspring to stress-induced brain alterations.^12^ Despite a large body of research indicating a potential correlation between prenatal stress and neurodevelopmental disorders in children, the underlying mechanisms remain poorly understood.^11^^,^^13-16^ Given the increase in the prevalence of prenatal stress,^17-20^ it is essential to uncover how stress during pregnancy affects offspring behavior and brain function at a neurobiological level.

The gut microbiota can be transmitted via maternal-neonatal vertical transfer,^21^ thereby affecting the development and function of the offspring's immune, metabolic, and nervous systems.^22^^,^^23^ Maternal stress and diet can functionally reshape microbial communities, affecting both the host and subsequent generations^23-26^ and potentially modulating disease manifestations in genetically susceptible individuals.^27^ With mounting evidence supporting the existence of the gut–brain axis,^28^^,^^29^ there is now emerging research on the crucial role of the gut microbiota in Impairment of social behavior and ASD.^30-32^ Autism is often accompanied by intestinal dysfunction and altered gut microbiome composition.^33^ Accordingly, fecal microbiota transplantation (FMT) from ASD donors was found to induce ASD behavioral phenotypes in the recipient mice.^30^ Given the increasing clinical evidence demonstrating the crucial role of gut microbiota in autism.^34-36^ we hypothesize that maternal stress-induced disruption of the gut microbiota in offspring may be a key factor contributing to the increased risk of social behavior impairment in offspring. However, the mechanisms via which maternal stress during pregnancy might affect an offspring's brain development and function via modulation of the gut microbiota are still unclear.

Here, we report that stress during pregnancy in rats induces social deficits through gut microbiome alterations in the offspring. Importantly, these stress-induced microbiota changes were found to impair neuroadaptation in the mesolimbic dopamine reward system (specifically the ventral tegmental area [VTA]). Interestingly, supplementation with *Limosilactobacillus reuteri* resulted in partial amelioration of maternal stress-induced social deficits in the offspring through vagus nerve-mediated modulation of oxytocin levels.

## 2. Materials and methods

### 2.1. Animals

The animals used in this study were purchased from the Animal Experiment Center of Central South University and were housed at the same center under a 12-h light/dark cycle with ad libitum access to food and water. The study protocol was approved by the Animal Ethics Committee of Central South University (Approval No.: CSU-2024-0266).

The prenatal stress (PS) model was established using Sprague-Dawley female rats aged over 9 weeks and that had never given birth. To induce prenatal stress, pregnant rats were placed in transparent plastic restrainers three times daily for 1 h each, from gestational days 16 to 20. To avoid the influence of factors such as the hormonal cycle on behavioral outcomes, only male offspring were selected for this study.

### 2.2. Groups

The detailed experimental groupings for each animal study are provided in the Supplemental Experimental Procedure.

### 2.3. Fecal microbiota transplantation

To deplete the gut microbiota, PS offspring were given water containing a broad-spectrum antibiotic mixture from postnatal day 21 (P21) to P27. The antibiotics included vancomycin hydrochloride (0.125 mg/mL/d; Macklin, C15668820), metronidazole (0.25 mg/mL/d; Solarbio, 443-48-1), neomycin sulfate (0.25 mg/mL/d; Solarbio, N8090), and ampicillin (0.25 mg/mL/d; Solarbio, A6920). Fresh fecal samples were collected from donor mice (control group offspring), homogenized with sterile PBS, then centrifuged at 4 °C, 1000g for 3 minutes. The supernatant was collected and diluted with sterile PBS to 5 × 10⁹ CFU/mL. FMT was performed in germ-free PS recipient rats at P28, P35, P42, and P49, with 1 mL of the solution. Fecal samples were collected from the rats before the first transplantation (P28) and one week after the final transplantation (P56) for 16S rRNA sequencing.

### 2.4. L-368899 administration

The oxytocin antagonist (L-368,899 hydrochloride; MCE) was dissolved in saline to a concentration of 5 mg/mL. It was administered intraperitoneally at a dose of 5 mg/kg 30 min before the three-chamber social behavior test.^37^^,^^38^

### 2.5. Immunofluorescence analysis

The detailed protocol for the immunofluorescence experiments is comprehensively described in the Supplemental Experimental Procedure.

### 2.6. Electrophysiology analysis

Electrophysiological recordings were performed in coronal brain slices (250 µm thick) containing the VTA during ZT09-12. Sprague-Dawley rats were sacrificed by decapitation after they were deeply anesthetized with sodium pentobarbital (50 mg/kg, i.p.). The brains were rapidly extracted and immersed in ice-cold choline-based artificial cerebrospinal fluid (ACSF) containing 120 mM choline chloride, 2.4 mM KCl, 7 mM MgCl₂·6H₂O, 0.5 mM CaCl₂, 1.25 mM NaH₂PO₄·2H₂O, 5 mM sodium ascorbate, 3 mM sodium pyruvate, 26 mM NaHCO₃, and 25 mM glucose (pH 7.2–7.3). Slices were prepared using a vibratome (7000 smz-2; Campden Instruments) with the tissue immersed in the ice-cold ACSF; the slices were immediately transferred to a recovery chamber containing normal ACSF (124 mM NaCl, 2.883 mM KCl, 26  mM NaHCO₃, 1.25  mM NaH₂PO₄·2H₂O, 10 mM glucose, 2 mM CaCl₂, and 1.2 mM MgCl₂·6H₂O, pH 7.2–7.3). The slices were maintained at 35.5 °C for 45–60 min and then kept at room temperature until the electrophysiological recordings. All the solutions were continuously oxygenated with 95% O₂/5% CO₂, and the slices were perfused with normal ACSF at 34–35 °C (2 mL/min) during the recordings.

For whole-cell recordings, patch pipettes (4–7 MΩ) were filled with an internal solution containing 140 mM K-gluconate, 3 mM KCl, 2 mM MgCl₂·6H₂O, 0.2 mM EGTA, 10 mM HEPES, and 2 mM Na₂ATP (285–295 mOsm/L, pH 7.2–7.25). The ACSF bath contained 100 μM of picrotoxin as a blocker of GABA_a_ receptors. VTA dopamine neurons were visually identified using IR-DIC microscopy (BX-51WI; Olympus), with the medial terminal nucleus and the medial lemniscus as anatomical landmarks. The neurons were confirmed based on these electrophysiological characteristics: (1) a spontaneous firing rate of 1–5 Hz with an action potential width > 1 ms, (2) membrane capacitance > 28 pF, and (3) the presence of Ih currents (>150 pA leakage current) upon hyperpolarizing steps from −40 to −120 mV. Recordings were performed using a MultiClamp 700B amplifier with signals acquired via Digidata 1550B and the pClamp 10.6 software.

AMPAR/NMDAR ratios were determined by voltage clamping of neurons at + 40 mV (holding current < 200 pA). Bipolar stimulating electrodes were placed 50–150 µm lateral to the recorded VTA neuron (at 0.05 Hz stimulation) to evoke excitatory postsynaptic currents (EPSCs). After recording dual-component EPSCs in picrotoxin-containing ACSF, the slices were perfused with DL-AP5 (100 µM) for 10 min to isolate AMPAR-mediated currents. NMDAR components were obtained by offline subtraction, and the AMPAR/NMDAR ratio was calculated from peak amplitude measurements.^39^ For detailed protocols of dopamine immunohistochemical staining, please refer to the Supplemental Experimental Procedure.

### 2.7. Bilateral subdiaphragmatic vagotomy

The detailed surgical protocol for bilateral subdiaphragmatic vagotomy is described in the Supplemental Experimental Procedure.

### 2.8. Culture of *L. reuteri* and its administration in experimental rats

The *L. reuteri* strain *ATCC-PTA-6475* was provided by Xiamen Chengge Biotechnology Co., Ltd. and cultured in MRS broth medium. After anaerobic growth at 37 °C for 48 h, the bacteria were collected by centrifugation (2000  rpm, 4 °C, 10 minutes), washed three times with sterile PBS, and stored in sterile glycerol at −80 °C until use. The bacterial concentration was determined using a standard plate count method. Briefly, a 10-fold gradient dilution of the bacterial suspension was prepared with sterile PBS, and 100 µL of the corresponding concentration of the bacterial dilution was plated on agar. After anaerobic incubation at 37 °C for 48 h, the number of colonies on plates containing 30–300 colonies was counted. This concentration was considered the final treatment concentration of 10⁸ CFUs, and the bacterial suspension was adjusted to this concentration. Intervention started at weaning (21 d) and continued for one month. During treatment, sterile PBS (solvent control) or *L. reuteri* (1*10⁸ organisms/rat/d) was added to the drinking water. Three rats were grouped together, and the water was replaced daily to minimize variation in dosage. Rats had free access to the water throughout the experiment. Fecal samples for 16S rRNA analysis were collected at the end of the treatment.

### 2.9. 16 s rRNA gene sequencing

Postnatal weeks 4–8 represent a critical window for neural development and gut microbiota establishment. Therefore, we selected this time frame to implement interventions including cohousing, FMT, and *L. reuteri* supplementation. Following the completion of the intervention (8 weeks postnatal), we evaluated the effects of each intervention factor on the gut microbiota via 16S rRNA sequencing. Total genomic DNA was extracted from fecal samples using the FastPure Stool DNA Isolation Kit (MJYH, Shanghai, China) according to the manufacturer's instructions. The integrity of extracted DNA was verified by 1% agarose gel electrophoresis, while concentration and purity were measured using a NanoDrop2000 spectrophotometer (Thermo Scientific, USA). The V3-V4 hypervariable regions of bacterial 16S rRNA genes were amplified from 10 ng template DNA using barcoded primers 338F (5′-ACTCCTACGGGAGGCAGCAG-3′) and 806 R (5′-GGACTACHVGGGTWTCTAAT-3′) in 20 μL reaction volumes containing 4 μL 5 × TransStart FastPfu buffer, 2 μL 2.5 mM dNTPs, 0.8 μL each primer (5 μM), 0.4 μL TransStart FastPfu DNA polymerase, with the following cycling parameters on an ABI GeneAmp® 9700 system: 95 °C for 3 min; 27 cycles of 95 °C for 30 s, 55 °C for 30 s, and 72 °C for 30 s; final extension at 72 °C for 10 min; hold at 4 °C. PCR products were size-selected by 2% agarose gel electrophoresis, purified using a PCR Clean-Up Kit (YuHua, China), and quantified with Qubit 4.0 (Thermo Fisher Scientific). Library preparation was performed using the NEXTFLEX Rapid DNA-Seq Kit through: (1) adapter ligation, (2) magnetic bead-based removal of self-ligated adapters, (3) PCR enrichment, and (4) final library purification with magnetic beads. Sequencing was conducted on the Illumina Nextseq2000 platform (Majorbio, Shanghai), with all bioinformatics analyzes performed on the Majorbio Cloud Platform (https://cloud.majorbio.com).

### 2.10. Three-chamber social interaction test

We conducted three-chamber social interaction tests at the end of various interventions (such as FMT, cohousing experiments, and *L. reuteri* intervention), specifically at the eighth week after birth. The three-chamber social interaction test for assessing sociability and social novelty preference was conducted as follows: The first step was a 10-min habituation period where the rat could freely explore the entire chamber, which was composed of a plexiglass arena (120 cm long × 48 cm wide × 40 cm high) that was divided into three interconnected chambers of equal size. The subject then underwent two consecutive 10-min test phases. In the sociability phase, the test rat could choose between interacting with an empty wire cage (10 cm diameter × 20 cm height) or an identical cage containing an unfamiliar, age- and sex-matched conspecific (Rat 1), with the cage positions (left/right chambers) interchanged across trials. All interactions (such as sniffing and crawling) and chamber occupancy were automatically tracked using the Smart 3.0 video tracking system. For the social novelty preference test, a second unfamiliar rat (Rat 2) was introduced to the previously empty cage, while the now-familiar Rat 1 remained in its original location. As before, all interactions (sniffing and crawling) and chamber occupancy were automatically tracked using the Smart 3.0 video tracking system.

### 2.11. Whole genome shotgun sequencing

We performed metagenomic sequencing on fecal samples collected from male rats at weaning (postnatal day 21). Genomic DNA was extracted from fecal samples using the FastPure Stool DNA Isolation Kit (Magnetic bead, MJYH, Shanghai, China), followed by quality assessment including DNA concentration/purity measurement and integrity verification via 1% agarose gel electrophoresis. The qualified DNA was sheared to approximately 350 bp fragments using Covaris M220 (Genes Company, China) and subjected to paired-end (PE) library construction with NEXTFLEX Rapid DNA-Seq Kit (Bioo Scientific, USA). Metagenomic sequencing was performed on Illumina NovaSeq™ X Plus platform (Illumina, USA) through Majorbio Pharmaceutical Technology (Shanghai) using the following bridge amplification workflow: (1) library molecules hybridized to flow cell primers via complementary bases for initial template fixation; (2) “bridge” formation through random hybridization of molecule ends to adjacent primers; (3) cluster generation via PCR amplification; (4) linearization of DNA amplicons into single strands; (5) cyclic nucleotide incorporation using engineered DNA polymerase and fluorescently labeled dNTPs (one base per cycle); (6) laser-based fluorescence detection for sequence determination; and (7) chemical cleavage of fluorescent/termination groups to enable subsequent base incorporation.

### 2.12. Statistical analysis

Data are expressed as mean ± standard error of the mean. Behavioral data were analyzed using two-tailed unpaired Student's *t*-tests or one-way ANOVA, followed by Bonferroni correction for multiple comparisons. All the statistical computations and graphical representations were generated using GraphPad Prism 6 (GraphPad Software). Microbiome analysis of 16S rRNA gene sequencing and whole-genome shotgun sequencing data was performed using the Majorbio Cloud Platform (https://v.majorbio.com/project-center/overview), built for community profiling and visualization.

## 3. Results

### 3.1. Prenatal stress impairs social behavior in offspring

**Figure 1.**
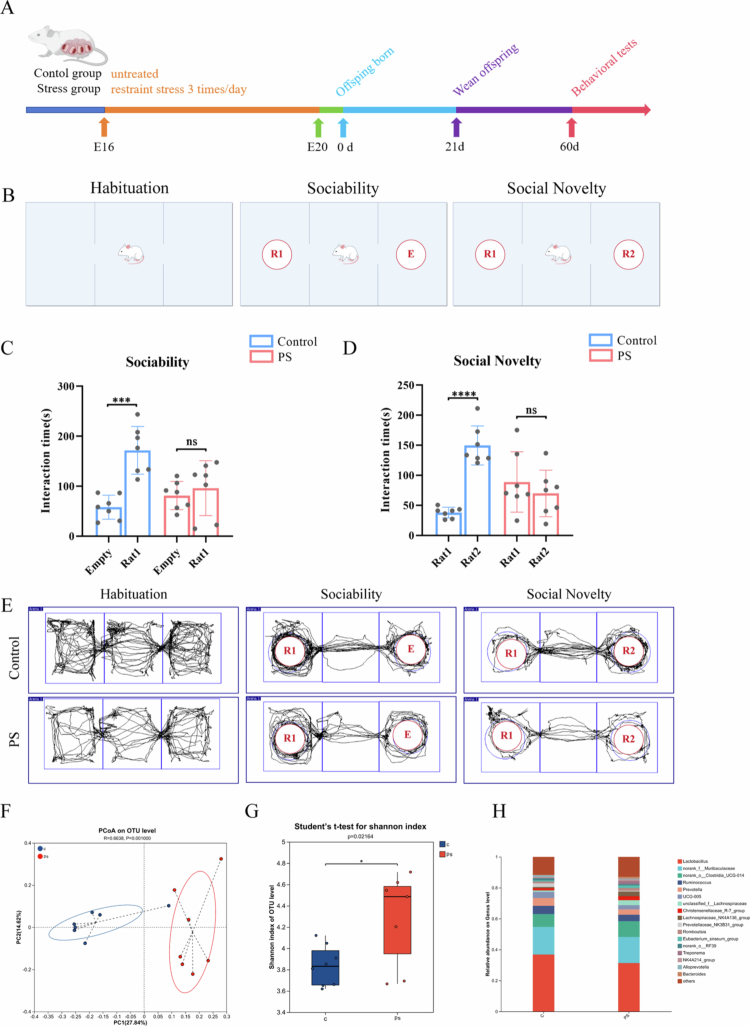
Impairment of social behaviors and gut microbiota in PS offspring. (A) Experimental workflow. (B) Schematic of the three-chamber social behavior test. (C) Sociability test: Control offspring spent more time interacting with Rat 1 than with an empty cage, whereas PS offspring showed no preference for either. (D) Social novelty test: The controls preferred novel Rat 2 over familiar Rat 1, while the PS offspring exhibited no discrimination between the two. (E) Representative movement trajectories of control and PS offspring during the three-chamber social behavior test. (F) PCoA of gut microbiota (weighted UniFrac values) revealed differential clustering between control and PS offspring. (G) Alpha diversity (Shannon index) was significantly elevated in PS offspring. (H) Relative abundances of gut microbiota at the genus level across all groups. Plots show mean ± SEM.

To investigate the effects of prenatal stress on the social behavior of offspring, we first established a reliable prenatal stress model. Restraint stress in pregnant rats during gestational days 16–20 has been shown to impair cognitive function^40^ and fear extinction in adult offspring.^41^ Given that prenatal stress increases the risk of neurodevelopmental disorders, including ASD in offspring, we compared social behavior between adult offspring from the PS group and the control group (Figure 1A). First, we found that the PS offspring exhibited significantly reduced reciprocal social interactions compared to the control offspring (Supplementary Figure 1A–C, *n* = 5, interaction time: t = 8.995, *P* < 0.0001; contact duration: t = 4.586, *P* < 0.01). Subsequently, the three-chamber social interaction test was used to assess sociability and social novelty preference: (1) sociability was evaluated by comparing the time spent interacting with an empty wire cage versus a wire cage containing another rat and (2) preference for social novelty was assessed by measuring the time spent interacting with a familiar rat versus a novel, unfamiliar rat (Figure 1B). During the acclimation phase of the three-chamber social interaction test, the total distance traveled by rats in each group was measured. The results showed no significant difference in the total distance traveled among all groups (Supplementary Figure 1D, *n* = 7, t = 0.4339, *P* = 0.672). In the three-chamber test, when confronted with an empty chamber and a conspecific (rat 1), rats in the control group exhibited a clear preference for interacting with the conspecific (t = 5.634, *P* < 0.001), which reflects their normal sociality (Figure 1C). When simultaneously exposed to a familiar conspecific (rat 1) and an unfamiliar conspecific (rat 2), rats in the control group spent significantly more time interacting with rat 2 (t = 8.772, *P* < 0.0001), indicative of their robust preference for social novelty (Figure 1D). In contrast, rats in the PS group showed no significant difference in interaction time between the empty chamber and the conspecific (t = 0.6386, *P* = 0.5351). Furthermore, no significant difference was observed in the PS group regarding the interaction time between the familiar conspecific and the unfamiliar conspecific (t = 0.7909, *P* = 0.4443). These findings suggest that prenatal stress leads to impairments in social competence and social novelty preference in rats (Figure 1C–D, *n* = 7). Taken together, these findings indicate that maternal stress during pregnancy leads to impaired social behaviors in offspring.

### 3.2. Prenatal stress alters the balance of gut bacteria in offspring

Prenatal stress alters the maternal gut microbiota,^40^ which can be vertically transmitted to offspring through birth and lactation^21^ Given the well-documented role of gut microbiota in ASD,^30^^,^^42^^,^^43^ we hypothesized that these stress-induced microbial disruptions may contribute to the impaired social behavior observed in the offspring. In order to explore this hypothesis, we investigated whether prenatal stress affects the gut microbiota of offspring by using 16S rRNA gene sequencing to analyze the gut microbial composition of adult offspring. The *β*-diversity metric used for generating the principal coordinate analysis (PCoA) plot in this study was calculated based on the weighted UniFrac distance, which evaluates the microbial community structure by accounting for the relative abundances of operational taxonomic units (OTUs). Statistical analysis revealed a significant difference in the bacterial community composition between the offspring of the control group and those of the prenatal stress group (Figure 1F, *P =* 0.001). In addition, compared with the control group, we found that the *α*-diversity of the offspring in the prenatal stress group was significantly increased (Figure 1G, *P* = 0.02164), which is consistent with previous findings reporting altered maternal gut microbiota under stress conditions.^44^ At the genus level, we observed that the relative abundance of *Lactobacillus* in the offspring of the prenatal stress group was lower than that in the control group (Figure 1H). These findings are in alignment with the existing literature^11^^,^^45^ and demonstrate that PS induces significant changes in the composition and diversity of the gut microbiota in rat offspring.

### 3.3. Gut microbiota-mediated social dysfunction in PS offspring

**Figure 2.**
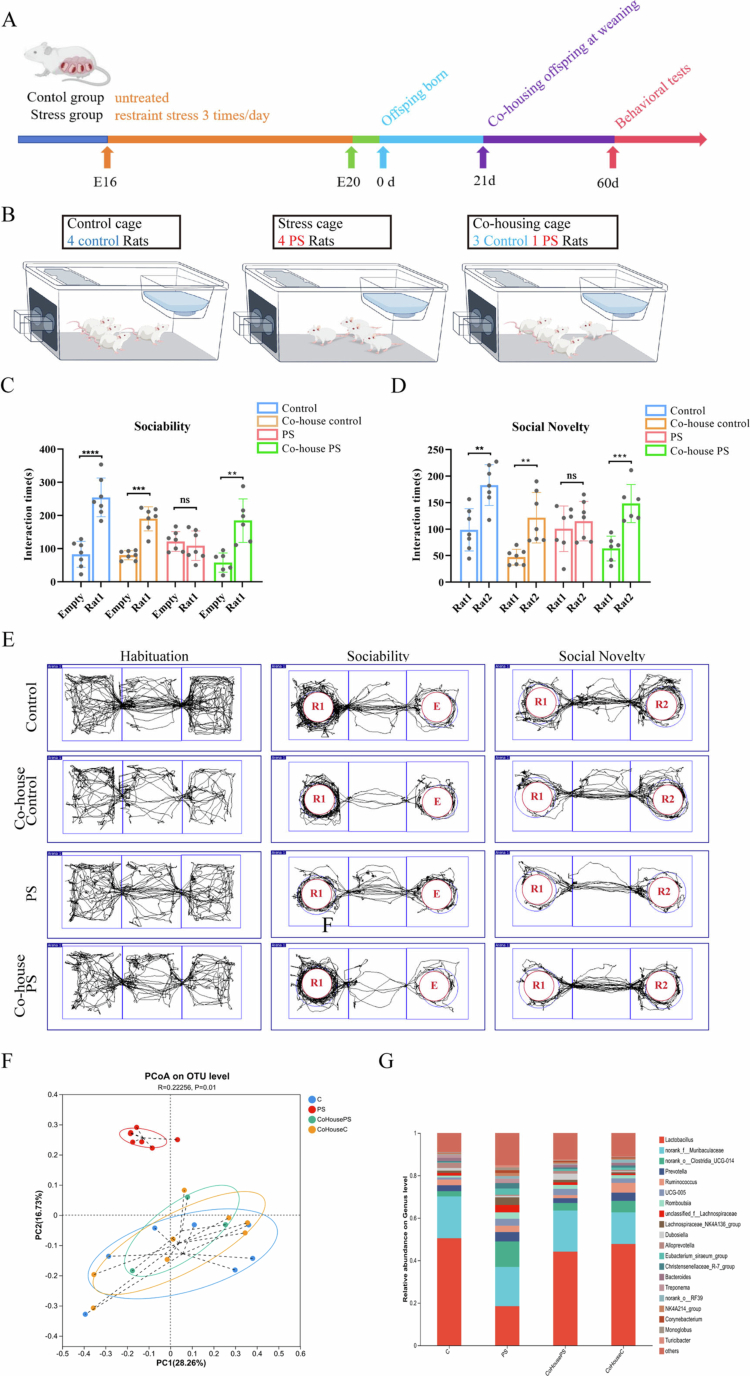
Partial rescue of social behavioral deficits in PS offspring cohoused with normal offspring. (A) Experimental workflow. (B) Schematic of the cohousing paradigm (3 control: 1 PS offspring per cage for 4 weeks). (C) Sociability test: cohousing PS increased interaction time with Rat 1 versus an empty cage, while PS offspring showed no preference for either. (D) Social novelty test: Cohoused PS offspring spent more time with novel Rat 2 than with familiar Rat 1, while PS offspring that were not cohoused showed no discrimination between the two. Plots show mean ± SEM. (E) Movement trajectories in the three-chamber social interaction test demonstrate restoration of social exploration in the cohoused PS offspring. (F) PCoA analysis revealed significant microbiota divergence between cohoused PS offspring and PS offspring that were not cohoused, with the cohoused samples clustering closer to the controls. (G) Relative abundances of gut microbiota at the genus level across all groups.

Studies have shown that rodents transmit microorganisms through the fecal-oral route, which results in shared microbial communities among cohoused animals.^24^^,^^46^ A critical period for neural development occurs between 4 and 8 weeks after birth in offspring.^24^ Accordingly, we tried to demonstrate the role of the gut microbiota in PS-induced social dysfunction in offspring by housing three weaned rats from the control group with one offspring from the PS group in the same cage for one month and examining their social behaviors with the help of a reciprocal social interaction task and the three-chamber social interaction test (Figure 2A, B). We found that co-housed PS offspring exhibited significantly increased reciprocal social interactions compared to PS offspring that were not co-housed with control offspring (Supplementary Figure 2A–C, *n* = 5, interaction time: F = 11.79, *P* < 0.001; contact duration: F = 33.40, *P* < 0.0001). During the acclimation phase of the three-chamber test, no significant difference was observed in the total distance traveled among the rats of each group (Supplementary Figure 2D, *n* = 7, F = 0.7978, *P* = 0.5072). Moreover, the cohoused PS offspring demonstrated improved sociability and social novelty preference compared to the PS offspring that were not cohoused (Figure 2C–E, *n* = 7). In the sociability test, rats in the control group showed a significant difference (*P* < 0.0001, t = 6.481); no significant difference was observed in rats in the PS group (*P =* 0.5341, t = 0.6402). For cohoused control rats, the difference was statistically significant (*P* < 0.001, t = 7.523), while co-housed PS rats also exhibited a significant difference (*P* < 0.01, t = 4.328). In the social novelty test, rats in the control group displayed a significant difference *P* < 0.01, t = 4.037); there was no significant difference in rats in the PS group (*P =* 0.5140, t = 0.6726). Cohoused control rats showed a statistically significant difference (*P* < 0.01, t = 3.934), and cohoused PS rats presented a highly significant difference (*P* < 0.001, t = 4.839). We also analyzed fecal samples from the offspring at 8 weeks and found that the gut microbiota composition of PS offspring cohoused with controls closely resembled that of the control offspring (Figure 2F, R = 0.22256, *P =* 0.01). The successful normalization of both social behaviors and microbiota composition in PS offspring with our cohousing paradigm (1 PS: 3 control) supports the notion that the fecal microbiota in PS offspring is deficient in key beneficial microbes necessary for typical social functioning (Figure 2G).

### 3.4. Fecal microbiota transplantation from control offspring to PS offspring effectively reverses their social deficits

**Figure 3.**
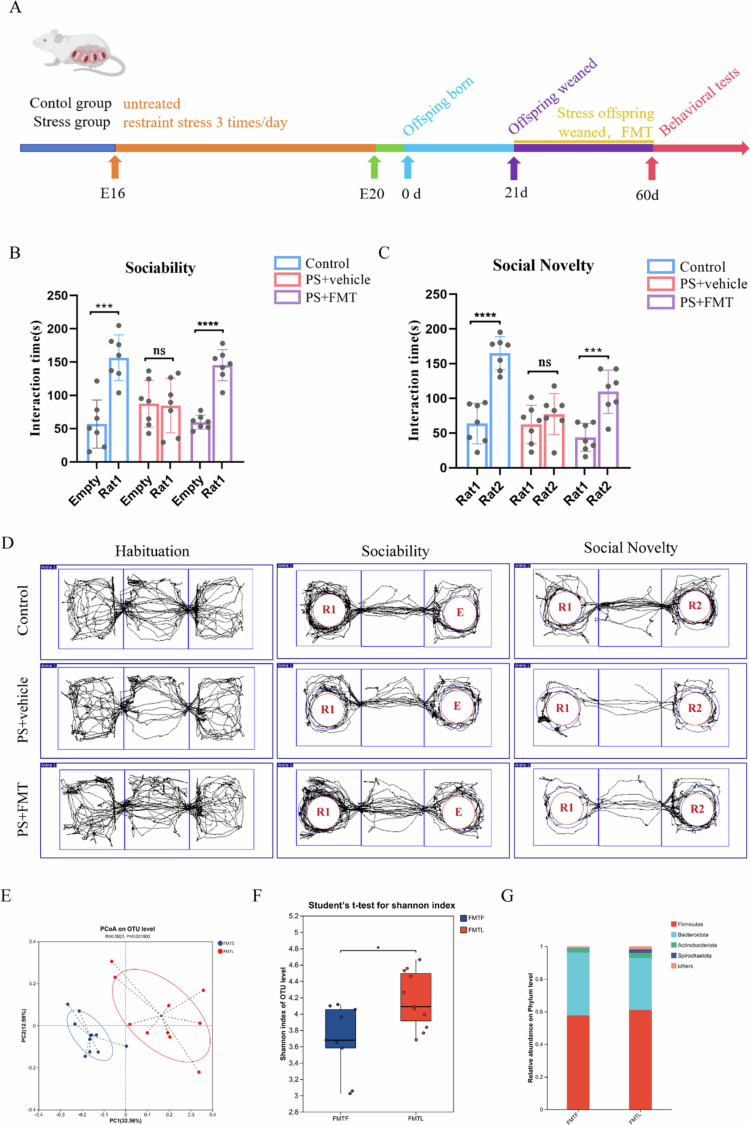
Partial amelioration of social behavioral deficits in PS offspring treated with fecal microbiota transplantation (FMT) with control offspring as donors. (A) Experimental workflow. (B) Sociability test: PS offspring receiving FMT showed significantly longer interaction time with Rat 1 compared to the empty cage, whereas untreated PS offspring displayed no preference for either. (C) Social novelty test: FMT-treated PS offspring preferentially interacted with novel Rat 2 over familiar Rat 1, while the untreated PS offspring showed no discrimination between the two. (D) Representative movement trajectories in the three-chamber social interaction test. (E) PCoA analysis revealed significant separation between the FMT-treated and untreated PS offspring. (F) Alpha diversity (Shannon index) was significantly higher in the FMT-treated than in the untreated PS offspring (*P = 0.02). (G) No significant differences in phylum-level composition were observed between the FMT and untreated PS groups. Plots show mean ± SEM.

To establish a causal relationship between gut microbiota and social deficits in PS offspring, we performed fecal microbiota transplantation (FMT) from control offspring to PS offspring during the critical neurodevelopmental window (postnatal weeks 4–8). The effects were examined by behavioral tests and 16S rRNA sequencing at week 8 (Figure 3A). PS offspring treated with FMT exhibited significantly increased reciprocal social interactions compared to non-treated PS offspring (Supplementary Figure 3A–C, *n* = 5, interaction time: F = 57.33, *P* < 0.0001; contact duration: F = 36.06, *P* < 0.0001). During the three-chamber habituation phase, no significant difference was detected in the total travel distance among the rats of each group (Supplementary Figure 3D, *n* = 7, F = 0.3116, *P* = 0.7362). Furthermore, FMT partially ameliorated the impairments in both sociability and social novelty preference observed in the PS offspring (Figure 3B–D, *n* = 7). In the sociability test: rats in the control group showed a significant difference (*P* < 0.001, t = 5.304); no significant difference was observed in rats in the PS group (*P* = 0.8958, t = 0.1338); rats in the FMT group exhibited a highly significant difference (*p* < 0.0001, t = 8.835). In the social novelty test: rats in the control group presented a highly significant difference (*P* < 0.0001, t = 7.129); there was no significant difference in rats in the PS group (*P* = 0.3458, t = 0.9814); rats in the FMT group showed a significant difference (*P* < 0.001, t = 6.674). With regard to the effects of FMT on the gut microbiota, PCoA revealed significant segregation of gut microbial composition in PS offspring after FMT as compared to before FMT (Figure 3E, *P* = 0.001). Moreover, after FMT, the PS offspring exhibited a significant increase in gut microbial *α*-diversity (Figure 3F, *P* = 0.01116). At the phylum level, the relative abundances of *Firmicutes* and *Spirochaetota* were significantly increased following FMT (Figure 3G). These data establish gut microbiota dysbiosis as a key link between PS and social dysfunction in offspring.

### 3.5. Supplementation with *L. reuteri* partially ameliorated the social deficits in PS offspring

**Figure 4.**
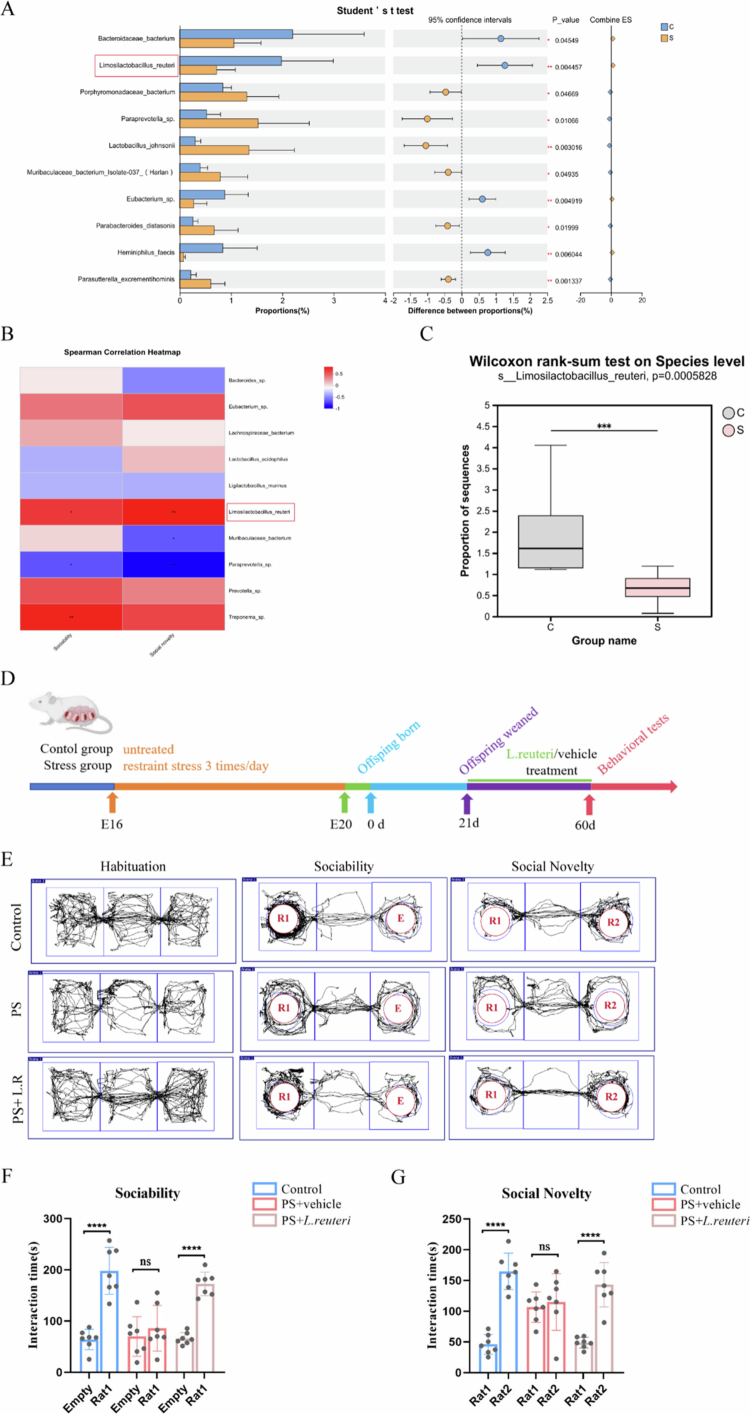
Partial rescue of social deficits in PS offspring supplemented with *L. reuteri*. (A) Differential abundance analysis of gut microbiota between control and PS offspring, demonstrating significantly reduced abundance of *L. reuteri* in the PS group. (B) Correlation analysis of behavioral results with metagenomic data: *L. reuteri* was identified as the top candidate positively associated with social performance. (C) Quantification of *L. reuteri* abundance showing reduced abundance in PS offspring compared to the controls. (D) Schematic of the *L. reuteri* intervention protocol. (E) Movement trajectories in the three-chamber social interaction test. (F) Sociability test: *L. reuteri*-supplemented PS offspring showed significantly longer interaction time with Rat 1 versus an empty cage, while the untreated PS group displayed no preference for either. (G) Social novelty test: *L. reuteri*-treated PS offspring preferentially interacted with novel Rat 2 over familiar Rat 1, but the untreated PS offspring did not display a preference for either. Plots show mean ± SEM.

To identify specific beneficial bacterial taxa that are impaired by prenatal stress, we performed metagenomic sequencing of fecal samples from both control and PS offspring. Vertical transmission of maternal microbiota occurs primarily during pregnancy, parturition, and lactation. After weaning, the gut microbiota of offspring is largely free from the direct influence of maternal feeding. This postweaning window is more suitable for reflecting the interference of PS on the process of maternal microbiota transmission, as well as the specific manifestations of such interference during the initial establishment stage of the offspring's gut microbiota. Therefore, the present study performed metagenomic sequencing on offspring samples at weaning (postnatal day 21). Comparative analysis revealed significant reductions in several putative beneficial microbes in the PS offspring, including *Bacteroidaceae bacterium*, *L. reuteri*, and *Eubacterium* (Figure 4A). To identify social behavior-associated microbial taxa, we performed correlation analyzes between species abundance and social behaviors dates in both the control and PS offspring. Notably, *L. reuteri* abundance showed significant positive correlations with both sociability and social novelty preference (Figure 4B, C).

Recent studies have demonstrated the efficacy of *L. reuteri* in improving social behavior.^47-49^ Accordingly, to establish the causal relationship between *L. reuteri* decrease and social deficits in PS offspring, we supplemented the PS offspring with *L. reuteri* from the time of weaning and assessed their social behavior as adult rats (Figure 4D). We found that *L. reuteri*-supplemented PS offspring exhibited significantly increased reciprocal social interactions compared to untreated PS offspring (Supplementary Figure 4A–C, *n* = 5, Interaction time: F = 64.73, *P* < 0.0001; Contact duration: F = 49.68, *P* < 0.0001). Furthermore, no significant difference was detected in the total travel distance of rats across all groups during the habituation phase of the three-chamber sociability test (Supplementary Figure 4D, *n* = 7, F = 0.1682, *P* = 0.8465). Consistent with our hypothesis, quantitative results from the three-chamber social interaction test demonstrated that compared with the PS group (t = 0.7192, *P* = 0.4858), rats in the PS + *L. reuteri* group exhibited a stronger preference for conspecifics over the empty chamber (Figure 4F, t = 10.99, *P* < 0.001). When simultaneously exposed to a familiar conspecific (rat 1) and an unfamiliar conspecific (rat 2) (Figure 4G), the PS + *L. reuteri* group spent significantly more time interacting with the unfamiliar conspecific (t = 6.727, *P* < 0.0001), whereas no such preference was observed in the PS group (t = 0.4324, *P* = 0.6731). These findings indicate that *L. reuteri* intervention significantly ameliorated the PS-induced impairment in social function (Figure 4E–G, *n* = 7). The results of principal coordinate analysis (PCoA) in the Lactobacillus reuteri-supplemented group showed that the gut microbial composition of L. reuteri-supplemented PS offspring was significantly segregated compared with that of PS offspring (Supplementary Figure 5A, *P* = 0.001). In addition, L. reuteri supplementation significantly altered the gut microbial *α*-diversity compared with PS offspring (Supplementary Figure 5B, *P* = 0.001). At the phylum level, we found that L. reuteri restored the gut microbial composition of prenatal stress offspring (Supplementary Figure 5C). At the genus level, we found that the relative abundance of Lactobacillus was higher in the PS + L. reuteri group than in the PS group (Supplementary Figure 5D). These findings suggest that a decrease in *L. reuteri* abundance may be a key factor related to the social behavioral deficits observed in PS offspring.

### 3.6. PS offspring exhibited significantly decreased oxytocin levels in the paraventricular nucleus (PVN)

**Figure 5.**
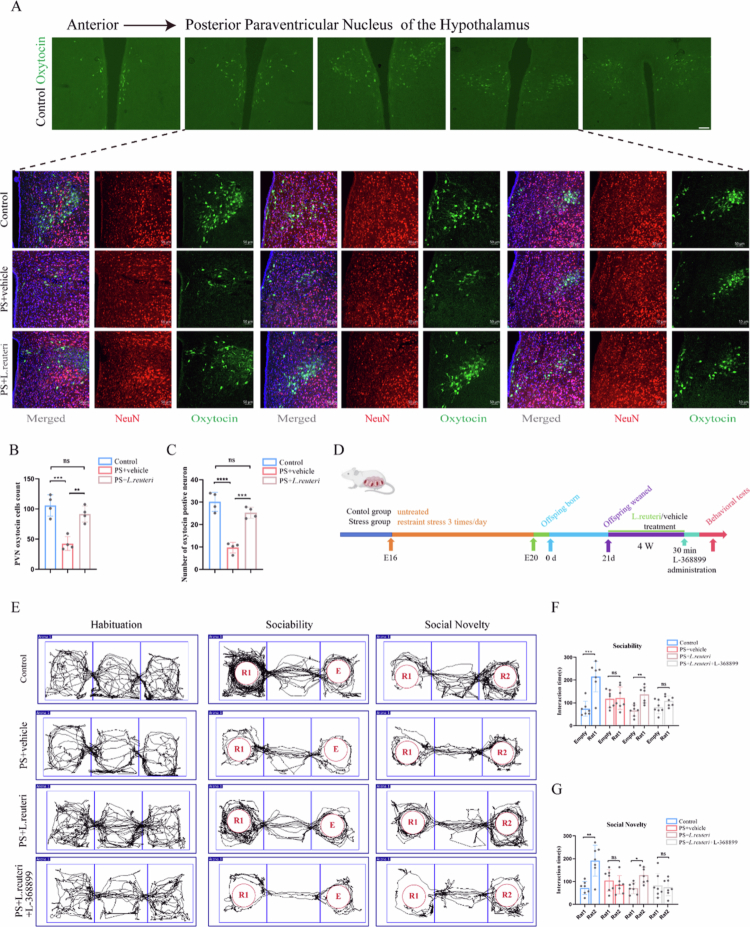
Oxytocin-dependent ameliorative effects of *Limosilactobacillus reuteri* on social deficits in PS offspring. (A) *L. reuteri* supplementation partially restored oxytocin (OXT) levels in the PVN of the PS offspring. (B) Quantification of OXT-immunoreactive cells in the PVN. (C) Quantification of OXT+  neurons in the PVN. (D) Experimental timeline of OXT receptor blockade: L-368,899 was administered 30 min before the behavioral tests. (E) Movement trajectories in the three-chamber social interaction test. (F) Sociability test: Treatment with *L. reuteri* alone rescued social novelty preference for Rat 1 over an empty cage, whereas co-treatment with *L. reuteri* and L-368,899 abolished this effect in the PS offspring. (G) Social novelty test: *L. reuteri* treatment restored social novelty preference, but coadministration of L-368,899 blocked this effect. Plots show mean ± SEM.

Experimental evidence has confirmed that *L. reuteri*-mediated enhancement of social behavior is dependent on oxytocin signaling.^48-50^ The hypothalamic PVN serves as the primary source of oxytocin secretion.^51^ Accordingly, we quantified oxytocin-expressing cells in the PVN in order to determine whether the social deficits caused by decreased *L. reuteri* in PS offspring are mediated through oxytocin signaling. Compared to the controls, the PS offspring exhibited a significant reduction in the number of both oxytocin-immunoreactive cells (*P* = 0.0004) and oxytocin-positive neurons (*P* < 0.0001). In contrast, PS offspring receiving *L. reuteri* supplementation showed a higher number of both oxytocin-immunoreactive cells (*P* = 0.0025) and oxytocin-positive neurons (*P* = 0.0002) than untreated PS offspring (Figure 5A–C). In summary, PS offspring exhibit reduced numbers of oxytocin-positive neurons in the PVN, and *L. reuteri* supplementation effectively restored oxytocinergic neuron populations in these animals.

### 3.7. Oxytocin receptor antagonism blocks the ameliorative effects of *L. reuteri* on social deficits in PS offspring

To further demonstrate that the rescuing effects of *L. reuteri* on social deficits in PS offspring are dependent on oxytocin signaling, we intraperitoneally injected the oxytocin receptor antagonist L-368899 into PS offspring supplemented with *L. reuteri* 30 min before behavioral testing (Figure 5D). Compared to PS offspring receiving only *L. reuteri* supplementation, those treated with both *L. reuteri* and the oxytocin receptor antagonist L-368899 exhibited significantly impaired sociability and social novelty preference (Figure 5E–G, *n* = 7). In the sociability test, rats in the control group showed a statistically significant difference (*P* < 0.001, t = 4.980); no significant difference was observed in rats in the stress group (*P* = 0.8804, t = 0.1526). For rats in the PS + *L. reuteri* group, a significant difference was detected (*P* < 0.01, t = 4.242), while rats in the PS + L-368899 group exhibited no statistical significance (*P* = 0.0693, t = 1.995). In the social novelty test, rats in the control group displayed a significant difference (*P* < 0.01, t = 4.291); there was no significant difference in the stress group (*P* = 0.4233, t = 0.8290). Rats in the PS + *L. reuteri* group showed a significant difference (*P* < 0.05, t = 3.559), whereas no significant difference was noted in the PS + L-368899 group (*P* = 0.7573, t = 0.3161). Thus, the therapeutic effect of *L. reuteri* supplementation on social deficits in PS offspring was abolished by oxytocin receptor antagonism.

### 3.8. PS offspring exhibit functional impairments in the mesolimbic dopamine reward circuitry

**Figure 6.**
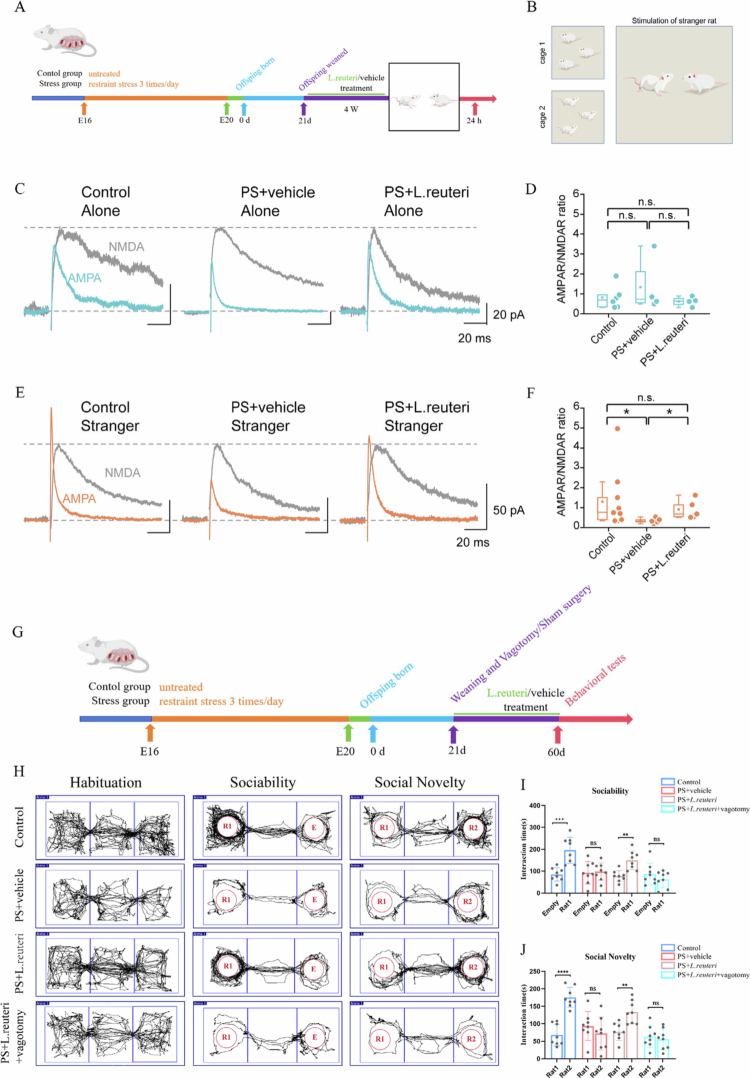
Vagus nerve-dependent mechanisms in the *Limosilactobacillus reuteri*-mediated amelioration of the dopaminergic reward circuitry and social deficits in PS offspring. (A) Electrophysiology workflow. (B) Schematic of social interaction behaviors before the electrophysiological measurements. (C, D) Social interaction with familiar rats failed to induce LTP in the control and PS offspring. (E, F) Novel social interaction induced LTP in the controls and *L. reuteri*-treated PS offspring, but this was not observed in the untreated PS group. (G) Surgical timeline for bilateral subdiaphragmatic vagotomy. (H) Movement trajectories in the three-chamber social interaction test. (I) Sociability test: *L. reuteri* treatment rescued social interaction preference, while vagotomy abolished this effect. (J) Social novelty: *L. reuteri* treatment restored social novelty preference, but this effect was negated by vagotomy. Plots show mean ± SEM.

The oxytocinergic neurons in the PVN can project to the ventral tegmental area (VTA), which is a key reward-processing region; this leads to the activation of neurons in the VTA and, thereby, modulates the interpretation of social cues.^52^ In rodents, social stimuli possess intrinsic rewarding properties and can induce synaptic potentiation in VTA dopamine neurons.^24^ Based on these findings, we investigated whether the reduction of oxytocin-positive neurons in the PVN of PS offspring impairs the dopaminergic reward circuitry of the VTA. To this end, we assessed whether direct social interactions can elicit synaptic long-term potentiation (LTP) in VTA dopaminergic (DA) neurons. We recorded the AMPAR/NMDAR ratios of glutamatergic excitatory postsynaptic currents (EPSCs) in both those offspring following 10-minute social interactions with either familiar or stranger rats (Figure 6A, B and Supplementary Figure 6). The control offspring exhibited LTP in VTA DA neurons following interactions with novel rats, but this was not observed after interactions with familiar rats. In contrast, the PS offspring showed impaired LTP induction with stranger rats. *L. reuteri*-supplemented PS offspring exhibited restored capacity for LTP triggered by novel social interactions (Figure 6C–F, control vs. PS: *P* = 0.0252, PS vs. *L. reuteri* + PS: *P* = 0.0420). These findings demonstrate that PS offspring develop functional deficits in the mesolimbic dopamine reward circuitry that can be partially rescued by *L. reuteri* supplementation.

### 3.9. *L. reuteri* ameliorates social deficits in PS offspring in a vagus nerve-dependent manner

The gut microbiota–brain axis operates bidirectionally, with gut bacteria generating signals that reach the central nervous system through circulatory pathways or vagal nerve transmission.^53^^,^^54^ Previous studies have shown that the vagus nerve can respond to specific bacterial strains and have suggested that *L. reuteri* may influence oxytocin secretion and social behavior via vagal pathways. Accordingly, we tried to determine whether *L. reuteri*-induced oxytocin release in the PVN is dependent on vagus nerve signaling by performing bilateral subdiaphragmatic vagotomy in PS offspring while administering *L. reuteri* supplementation. The control offspring underwent identical surgical procedures, except for vagal nerve transection (Figure 6G). Based on previous researches,^55^^,^^56^ the vagus nerve is a key pathway mediating the satiating effect of cholecystokinin-8 (CCK-8), but this effect is dependent on the integrity of the vagus nerve. Therefore, we utilized this characteristic to verify the effectiveness of vagotomy. The results of this study showed that compared with the sham-operated group, rats in the vagotomy group still exhibited a significant increase in food intake even after intraperitoneal injection of CCK-8 (*P* < 0.0001). This finding confirms that the vagotomy procedure was successfully performed with a definite blocking effect (Supplementary Figure 7A-B). Consistent with our hypothesis, quantitative results from the three-chamber social interaction test showed that, compared with the PS + *L. reuteri* + vagotomy group (t = 1.090, *P* = 0.2943), rats in the PS + *L. reuteri* group exhibited a stronger preference for conspecifics over the empty chamber (Figure 6I, t = 4.120, *P* < 0.001). When simultaneously exposed to a familiar conspecific (rat 1) and an unfamiliar conspecific (rat 2) (Figure 6J), the PS + *L. reuteri* group spent significantly more time interacting with the unfamiliar conspecific (t = 3.847, *P* < 0.001), whereas no such preference was detected in the PS + *L. reuteri* + vagotomy group (t = 0.4364, *P* = 0.6692). Collectively, these data demonstrated that *L. reuteri* supplementation rescued social deficits in PS offspring but failed to do so in the vagotomized PS offspring (Figure 6H–J, *n* = 7). Based on this observation, we next tried to determine whether *L. reuteri* regulates oxytocin secretion in the PVN via the vagus nerve by quantifying oxytocin-positive neurons in the PVN of offspring. We found that *L. reuteri* supplementation increased the number of oxytocin-positive neurons in the PVN of PS offspring; however, this effect was abolished in the vagotomized PS offspring (Supplementary Figure 8A-C). These results suggest that *L. reuteri* probably modulates oxytocin secretion in the PVN through vagus nerve-dependent mechanisms.

## 4. Discussion

PS is recognized as a potential risk factor for social behavior impairment.^57-59^ Previous studies investigating the association between PS and neurodevelopmental disorders (including ASD) have primarily focused on the dysregulation of the hypothalamic-pituitary-adrenal (HPA) axis.^60^ However, whether maternal stress impacts offspring neurodevelopment via other peripheral mechanisms, particularly the gut microbiota-brain axis, remains to be further elucidated. One of the key findings of this study is that prenatal stress reduces the abundance of Lactobacillus reuteri during a critical window of offspring neurodevelopment. Targeted supplementation with this bacterium not only effectively restores the gut microbial composition of prenatal stress offspring, but also reverses prenatal stress-induced social deficits. These findings further clarify the role of Lactobacillus reuteri in the crosstalk between gut microbiota and behavioral phenotypes. Collectively, our findings propose a potential preventive strategy: mitigating the adverse effects of prenatal stress on offspring neurodevelopment through early *L. reuteri* supplementation.

We established a prenatal restraint stress model to observe alterations in gut microbiota and behavioral phenotypes of offspring, aiming to investigate the role of gut microbiota in mediating the effects of prenatal stress on offspring behaviors. The results of this study demonstrated that prenatal stress-induced impairments in sociability and social novelty preference of offspring were closely associated with alterations in gut microbiota composition. Moreover, compared with offspring of rats in the control group, the offspring of the PS group exhibited increased gut microbial *α*-diversity. *α*-diversity (assessed by the Shannon index) refers to the species richness and evenness of the microbiota, and its value does not show a direct correlation with the state of intestinal health. We found that prenatal stress exposure induced a shift in the overall composition of the offspring gut microbiota, and the excessively elevated *α*-diversity might be attributed to the invasion of harmful microbial taxa or the dilution of beneficial microbial abundance.^61^ Studies on several diseases have reported both positive and negative associations of *α*-diversity with disease status. For instance, changes in *α*-diversity indices in Parkinson’s disease are not universally characterized by a decrease.^62^^,^^63^ In addition, a systematic review focusing on ASD and gut microbiota reported inconsistent trends in *α*-diversity indices: two studies demonstrated increased *α*-diversity, while six studies showed no significant changes.^64^ It has also been reported that maternal *α*-diversity increases under prenatal stress conditions.^44^ We further identified that multiple intrinsic and extrinsic/environmental factors can also contribute to variations in baseline microbial diversity, thereby leading to the instability of *α*-diversity measurements.

In our experiments, cohousing offspring of PS dams with offspring of control dams partially ameliorated the social deficits of the former. Although we tend to attribute this effect to microbiota sharing via the fecal-oral route, an alternative explanation must be ruled out: the introduction of new cage mates enhances social novelty stimulation, thereby improving behavioral performance. To verify this, we adopted two complementary strategies. First, gut microbiota profiling revealed that cohousing indeed drove the microbiota signature of PS offspring to converge toward that of control offspring. Second, we performed an independent FMT experiment and found that transplantation of feces from control rats alone was sufficient to significantly improve the social deficits of PS offspring. This result eliminated the confounding effect of social interaction per se and established the central role of gut microbiota in ameliorating PS-induced social impairments. This conclusion is also supported by the work of Vanessa et al., who similarly confirmed that cohousing can drive the convergence of gut microbiota and metabolic signatures.^65^

Interestingly, we selected Sprague-Dawley (SD) rats as the research subjects to investigate the role of gut microbiota in PS-induced behavioral abnormalities in offspring, based primarily on the following considerations. First, as a classic model organism, SD rats exhibit stronger physiological relevance to humans than mice,^66^^,^^67^ and the composition of their fecal microbiota is also significantly more similar to that of humans,^68^^,^^69^ which provides a crucial basis for cross-species translational research. Second, FMT experiments using germ-free rats have confirmed that most of the highly abundant microbes in the human gut can be successfully colonized in the rat gut.^70^ Furthermore, the relative abundances of certain key taxa in the human gut—such as *Clostridium cluster XI*, *Akkermansia*, and some species belonging to the *Lachnospiraceae* family—are highly similar between rats and humans, a feature that is absent in mice.^71^ Therefore, elucidating the mechanism underlying the ameliorative effect of *L. reuteri* on social behaviors using a rat model can provide more targeted evidence for clinical translation.

Vertical transmission of the maternal microbiota occurs primarily during the perinatal and lactation periods.^21^ After weaning (postnatal day 21, P21), the gut microbiota of rats is no longer directly affected by breast milk feeding,^72^ and postnatal weeks 4–8 (P4–P8) represent a critical window for both neurodevelopment and microbiota establishment in rats.^24^ Therefore, we performed metagenomic sequencing at P21 to accurately reflect the early shaping effect of the maternal microbiota on the offspring's microbiota; interventions were implemented during P4–P8 to achieve long-lasting effects on adult behaviors and microbial profiles. The findings of our study suggest that early-life supplementation with *L. reuteri* is a potential preventive strategy for alleviating the adverse impacts of PS on neurodevelopment. Meanwhile, our findings are highly consistent with clinical observations: studies have shown that offspring of mothers exposed to high levels of stress during pregnancy exhibit increased abundances of pathogenic bacteria (e.g., *Escherichia spp*.) and decreased abundances of beneficial bacteria (e.g., *Lactobacillus* and *Bifidobacterium*) in the gut.^73^ On this basis, we further employed metagenomic sequencing to precisely identify *L. reuteri* as a key species with significantly reduced abundance. Previously, Buffington et al. demonstrated that *L. reuteri* could alleviate maternal high-fat diet-induced social deficits in mice.^24^ Interestingly, our study extends the therapeutic potential of this bacterium to the prenatal stress model. These results indicate that multiple adverse maternal environmental factors (dietary alterations or psychological stress) may interfere with the vertical transmission of *L. reuteri* through a common mechanism, thereby impairing offspring health.

Our findings reveal how PS induces social behavioral abnormalities in offspring through the axis of *L. reuteri* depletion and impaired oxytocin signaling in the PVN. Administration of an oxytocin receptor antagonist completely abrogated the ameliorative effects of *L. reuteri*, confirming the critical role of the oxytocin pathway. Although intranasal oxytocin administration has been proven effective, it is associated with limitations in long-term interventions, including a short duration of action, poor brain penetration, and a high risk of receptor desensitization.^74-76^ In contrast, oral *L. reuteri* supplementation offers a noninvasive and convenient alternative that can sustainably elevate endogenous oxytocin levels, thereby holding greater potential for clinical translation. As a probiotic with FDA GRAS (generally recognized as safe) certification,^77^ the safety of *L. reuteri* has been extensively validated in humans, including infants. Future validation of its therapeutic efficacy in individuals with social behavior impairment via nonhuman primate studies or clinical trials will provide robust support for the development of novel therapeutic strategies.

Although we have demonstrated that *L. reuteri*-mediated regulation of oxytocin levels in the PVN is dependent on vagal nerve signaling, the precise underlying mechanism remains incompletely understood. Emerging evidence indicates that gut enteroendocrine cells (neuropod cells) possess neuroendocrine functions and are capable of forming direct synaptic connections with vagal afferent fibers.^78^ This raises an intriguing question worthy of further investigation: does *L. reuteri* modulate the signal transduction of gut enteroendocrine cells, thereby altering vagal afferent transmission to the brain? In-depth exploration of this “gut–brain” neural circuit will help elucidate the specific pathway through which microbial interventions regulate neurodevelopment. Previous therapeutic options for PS-induced neurological dysfunction have been relatively limited. While our research group recently proposed that prenatal probiotic supplementation can improve cognitive function in offspring,^40^ the prenatal intervention window is narrow and often difficult to implement prior to the onset of symptoms. The present study demonstrates that *L. reuteri* supplementation during childhood (corresponding to the juvenile period in rats) remains efficacious. This not only extends the intervention time window but also provides a safe and feasible remedial strategy for children already presenting with PS-associated social behavior impairment symptoms.

This study still has certain limitations. First, apart from *L. reuteri*, we have not yet explored in depth the potential contributions of other bacterial species with reduced abundances in the offspring of PS. Second, the specific molecular mechanism by which *L. reuteri* activates the vagus nerve remains to be further elucidated. Third, given that males are more sensitive to maternal stress and females exhibit periodic hormonal fluctuations, this study only analyzed the results from male offspring. Therefore, the conclusions of this study may not be generalizable to females. Future studies could focus on female offspring, as well as the detailed interactions between L. reuteri, intestinal enteroendocrine cells, and afferent vagus nerve pathways.

To summarize, through findings at four levels, this study confirms that the social deficits of offspring induced by prenatal stress are closely associated with gut microbiota dysbiosis: (1) PS leads to significant gut microbiota dysbiosis in offspring; (2) restoration of the microbiota via cohousing or FMT can partially ameliorate social deficits; (3) metagenomic analysis identifies *L. reuteri* as the key depleted species, and supplementation with this bacterium can restore social function; and (4) *L. reuteri* regulates oxytocin levels in the PVN via the vagus nerve, thereby improving the function of the dopaminergic reward circuit. These findings highlight the critical role of the gut microbiota–brain axis in the pathogenic mechanism of PS and clarify the important value of *L. reuteri* and the oxytocin pathway as potential therapeutic targets.

## Supplementary Material

Supplemental Experimental Procedures.docx Supplemental Experimental Procedures.docx

## Data Availability

The original sequencing data used in this study has been deposited in the NCBI database under accession code PRJNA1300300.
