# Supplementary material for: Limosilactobacillus reuteri normalizes gut microbiota dysfunction and social deficits of rat offspring associated with prenatal exposure to stress
Source: Gut Microbes. 2026 Mar 30;18(1):2649440. doi: 10.1080/19490976.2026.2649440 (PMC13037440; doi:10.1080/19490976.2026.2649440)
Supplement: Supplemental Experimental Procedures.docx [file KGMI_A_2649440_SM2199.docx]

**Supplemental experimental procedure**

**Groups**

Experiment 1

The pregnant rats were randomly allocated into either the control group or the prenatal stress (PS) group, with the PS group subjected to restraint stress during gestation. Subsequently, fecal samples were collected from the offspring at postnatal day 21 (P21) for metagenomic sequencing analysis. The offspring underwent behavioral testing and fecal 16S rRNA sequencing upon reaching adulthood.

Experiment 2

The offspring derived from normal pregnant rats and prenatal stress (PS)-exposed dams were allocated into four experimental groups: (1) Control offspring (from unstressed mothers), (2) PS offspring (from stressed mothers), (3) Co-housed Control offspring, and (4) Co-housed PS offspring. During postnatal weeks 4-8, we implemented a standardized co-housing paradigm where three Co-housed Control offspring were housed together with one Co-housed PS offspring per cage to facilitate microbial transfer. At 8 weeks of age, all subjects underwent behavioral assessments, followed by fecal sample collection for microbial community analysis through 16S rRNA gene sequencing.

Experiment 3

The offspring from normal pregnant rats and prenatal stress (PS)-exposed dams were divided into three experimental groups: (1) Control group, (2) PS group and (3) PS+FMT group (PS offspring receiving fecal microbiota transplantation from control donors). To prepare for microbiota manipulation, PS+FMT group were administered antibiotic-containing drinking water from postnatal day 21 (P21) to eliminate indigenous gut microbiota. Subsequently, fecal microbiota transplantation was performed at four critical time points (P28, P35, P42, and P50) to ensure stable microbial engraftment. Upon reaching adulthood, all rats underwent behavioral tests and fecal sample 16S rRNA gene sequencing.

Experiment 4

The offspring derived from normal pregnant rats and prenatal stress (PS)-exposed dams were systematically allocated into three experimental groups: (1) Control group, (2) PS group, and (3) PS+L.R group (PS offspring receiving *L. reuteri* supplementation). During postnatal weeks 4-8, the PS+L.R group was administered *L. reuteri* while control and PS groups received vehicle. Upon reaching adulthood, all rats underwent behavioral tests and fecal sample 16S rRNA gene sequencing.

Experiment 5

The offspring from normal pregnant rats and prenatal stress (PS)-exposed dams were allocated into four experimental groups: (1) Control group, (2) PS group, (3) PS+L.R group and (4) PS+L.R+L-368899 group. During the critical postnatal period of weeks 4-8, the PS+L.R+L-368899 group received daily co-administration of *L. reuteri* and L-368899 via oral gavage, while other groups received their respective interventions. Upon reaching adulthood, all subjects underwent behavioral tests and fecal sample 16S rRNA gene sequencing.

Experiment 6

The offspring of normal pregnant rats and prenatal stress-exposed pregnant rats were divided into the following groups: control group, PS offspring group, PS+L.R group, and PS+L.R+vagotomy group. In the PS+L.R+vagotomy group, bilateral vagotomy was performed on the PS offspring at 4 weeks of age, and from weeks 4 to 8, the PS offspring was supplemented with Limosilactobacillus reuteri (*L. reuteri*). Upon reaching adulthood, behavioral experiments and fecal 16S rRNA sequencing were conducted.

**Reciprocal Social Interaction**

Pairs of rats were introduced into a novel plexiglass arena (previously unhabituated) containing either a familiar cage-mate or an age-/sex-matched unfamiliar conspecific. Social interactions were quantified by measuring the duration of active social engagement (including mutual sniffing, allogrooming, and play behavior) during the observation period. To ensure objective assessment, all behavioral recordings were analyzed using Smart 3.0 automated tracking software, with the experimenter blinded to treatment group assignments throughout analysis phases.

**Dopaminergic neuron identification**

Following electrophysiological recordings, the 250-μm-thick brain slices were subjected to dopaminergic neuron identification through immunohistochemical staining. The slices were first incubated overnight at 4°C with primary antibodies against tyrosine hydroxylase (TH, 1:500 dilution; Proteintech, 25859-1-AP) to label dopaminergic neurons and anti-avidin-RB (1:1000 dilution; Millipore) to visualize the biocytin-filled recorded cells. After washing, the slices were then treated with Alexa Fluor 488-conjugated secondary antibody(Jackson ImmunoResearch, USA) for 1 hour in the dark to detect TH-positive neurons, while the recorded cells were visualized through the biocytin-avidin system. Nuclear counterstaining was performed using DAPI to identify cellular architecture. Finally, the colocalization of electrophysiologically recorded cells with dopaminergic neurons was confirmed through high-resolution imaging using a confocal microscope (Zeiss LSM800), enabling precise correlation between the functional properties obtained during recordings and the dopaminergic identity of the neurons.

**Validation of Vagotomy Completeness**

Before surgery, Rats were acclimated to a liquid diet (35% sweetened condensed milk solution) for two days and fasted overnight before the procedure. Anesthesia was induced using gaseous isoflurane (Henry Schein Animal Health) in an induction chamber. The surgical site was shaved and disinfected with three alcohol wipes before performing a midline laparotomy. The liver and small intestine were gently retracted to expose the stomach and lower esophagus. Both vagal trunks were carefully dissected from the esophagus, with all neural and connective tissues surrounding the esophagus (below the diaphragm) excised to ensure complete vagal branch transection. Sham-operated rats underwent identical procedures without vagal dissection. The abdominal muscle layer was closed with absorbable 5-0 Vicryl sutures (tapered needle), while the skin was approximated using non-absorbable 5-0 nylon or prolene sutures (cutting needle). Tissue adhesive was applied to create a waterproof seal over the incision. Extended-release buprenorphine (1 mg/kg) was administered subcutaneously 1 hour preoperatively, followed by a second dose 72 hours postsurgery. Postoperative recovery occurred in clean cages partially placed on heating pads, with animals returned to housing after regaining alertness without complications. To prevent ileus, mice received the liquid diet for 3 days postsurgery, followed by 2 days of nutritionally complete gel diet (Fanbo, Shanghai) before transitioning to regular chow. Daily monitoring continued for at least 1 week to ensure full recovery, with particular attention to weight stabilization and normal behavioral patterns^[1]^.

To validate the completeness of vagotomy, we performed a satiety test based on cholecystokinin-8 (CCK-8) response, as vagally-mediated satiety is specifically induced by CCK-8 administration^[2]^. After 20 hours of food deprivation, both sham-operated and vagotomized rats were individually housed and intraperitoneally injected with CCK-8 (8 μg/kg body weight; Sigma-Aldrich) dissolved in 0.1% PBS. Food intake was monitored for 2 hours post-injection by weighing food pellets before and after testing. The vagotomy verification was conducted at least two weeks after the final experimental treatment, accounting for potential probiotic-mediated modulation of CCK-8's satiety effects^[3,4]^, which could otherwise confound the interpretation of vagal integrity.

**Immunofluorescence**

Rats were deeply anesthetized with isoflurane and transcardially perfused with 200 mL of 0.9% saline followed by 200 mL of 4% paraformaldehyde (PFA). Brains were post-fixed in 4% PFA at 4°C overnight, dehydrated in sucrose, and embedded in OCT compound (Sakura). Coronal sections (30 μm thickness) were cut using a cryostat (Leica), collected in ice-cold PBS, and processed for immunofluorescence. Sections were blocked with 10% normal goat serum for 1 hr, then incubated for 24 hr at 4°C with primary antibodies (rabbit anti-oxytocin, ImmunoStar #20068, 1:2000; mouse anti-NeuN, Millipore #MAB377, 1:2000) in blocking solution. After three washes with 0.3% Triton X-100 in 0.1M PB, sections were incubated for 1 hr at room temperature (protected from light) with secondary antibodies (goat anti-rabbit Alexa Fluor 488, ThermoFisher #A-11034, 1:1000; goat anti-mouse Alexa Fluor 594, ThermoFisher #A-11032, 1:1000). Following PBS washes, sections were mounted with DAPI-containing medium and imaged by confocal microscopy.

For quantitative immunofluorescence analysis, 24 consecutive brain sections (30 μm thickness) spanning the rostrocaudal extent of the paraventricular nucleus (PVN) were collected from each mouse. Using ImageJ software (NIH), we systematically counted oxytocin (OXY)-immunopositive cells and NeuN-positive neurons in each section. The mean values of OXY⁺ and NeuN⁺ cell counts across all 24 sections were calculated to represent each animal's oxytocin neuron density and total neuronal population in the PVN region.

[1] Sgritta M, Dooling S W, Buffington S A, et al. Mechanisms Underlying Microbial-Mediated Changes in Social Behavior in Mouse Models of Autism Spectrum Disorder[J]. Neuron, 2019, 101(2): 246-259.e6.

[2] Owyang C, Heldsinger A. Vagal control of satiety and hormonal regulation of appetite[J]. J Neurogastroenterol Motil, 2011, 17(4): 338-48.

[3] Falcinelli S, Rodiles A, Unniappan S, et al. Probiotic treatment reduces appetite and glucose level in the zebrafish model[J]. Sci Rep, 2016, 6: 18061.

[4] Fåk F, Bäckhed F. Lactobacillus reuteri prevents diet-induced obesity, but not atherosclerosis, in a strain dependent fashion in Apoe-/- mice[J]. PLoS One, 2012, 7(10): e46837.

**Supplementary Data**


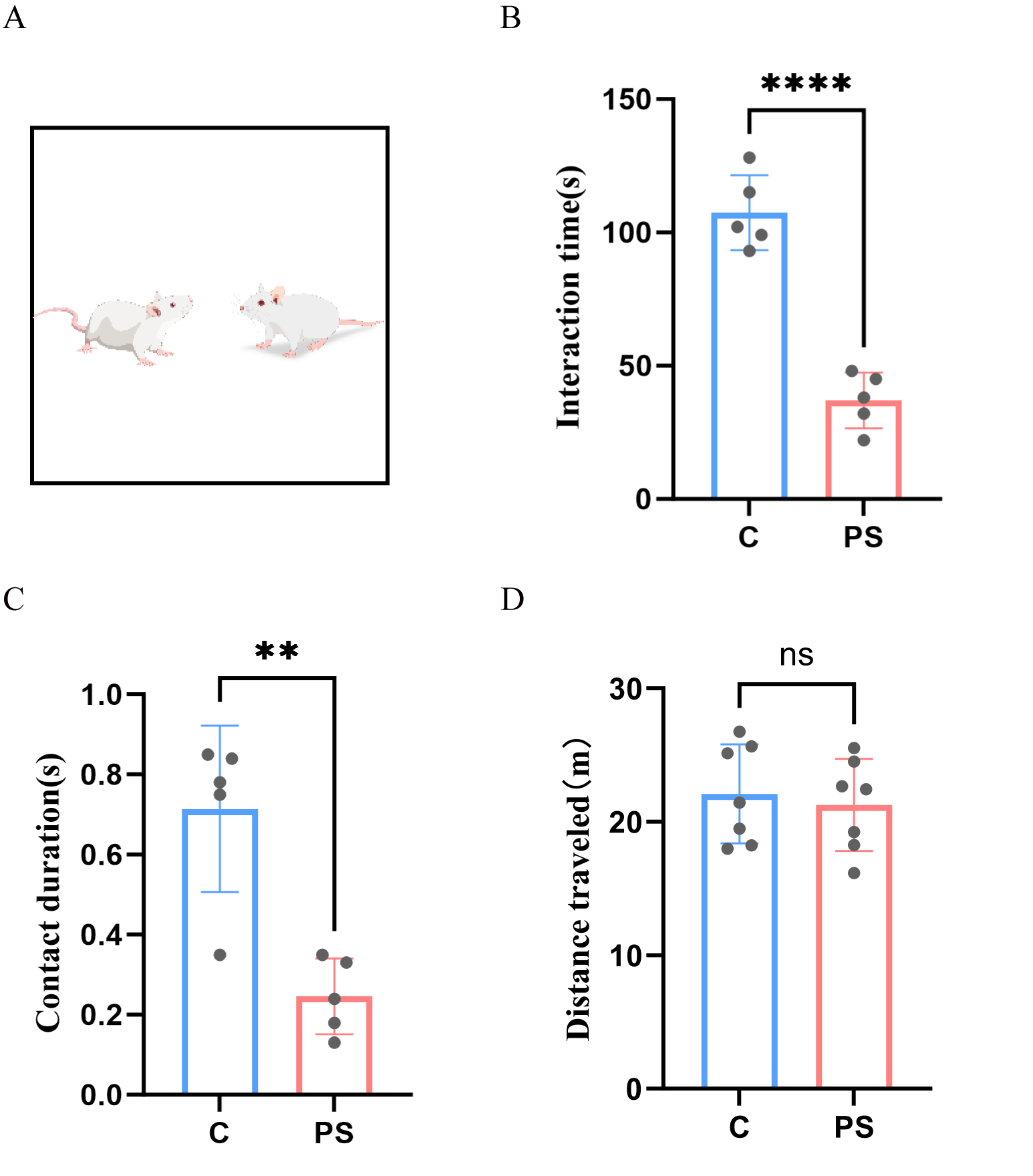


Supplementary Figure 1

A Schematic of the reciprocal social interaction task.

B-C PS offspring exhibited significantly reduced reciprocal social interactions compared to control groups (n = 5).

D The total distance moved by rats in the empty box period of the three-box social experiment within 10 minutes (n = 7).


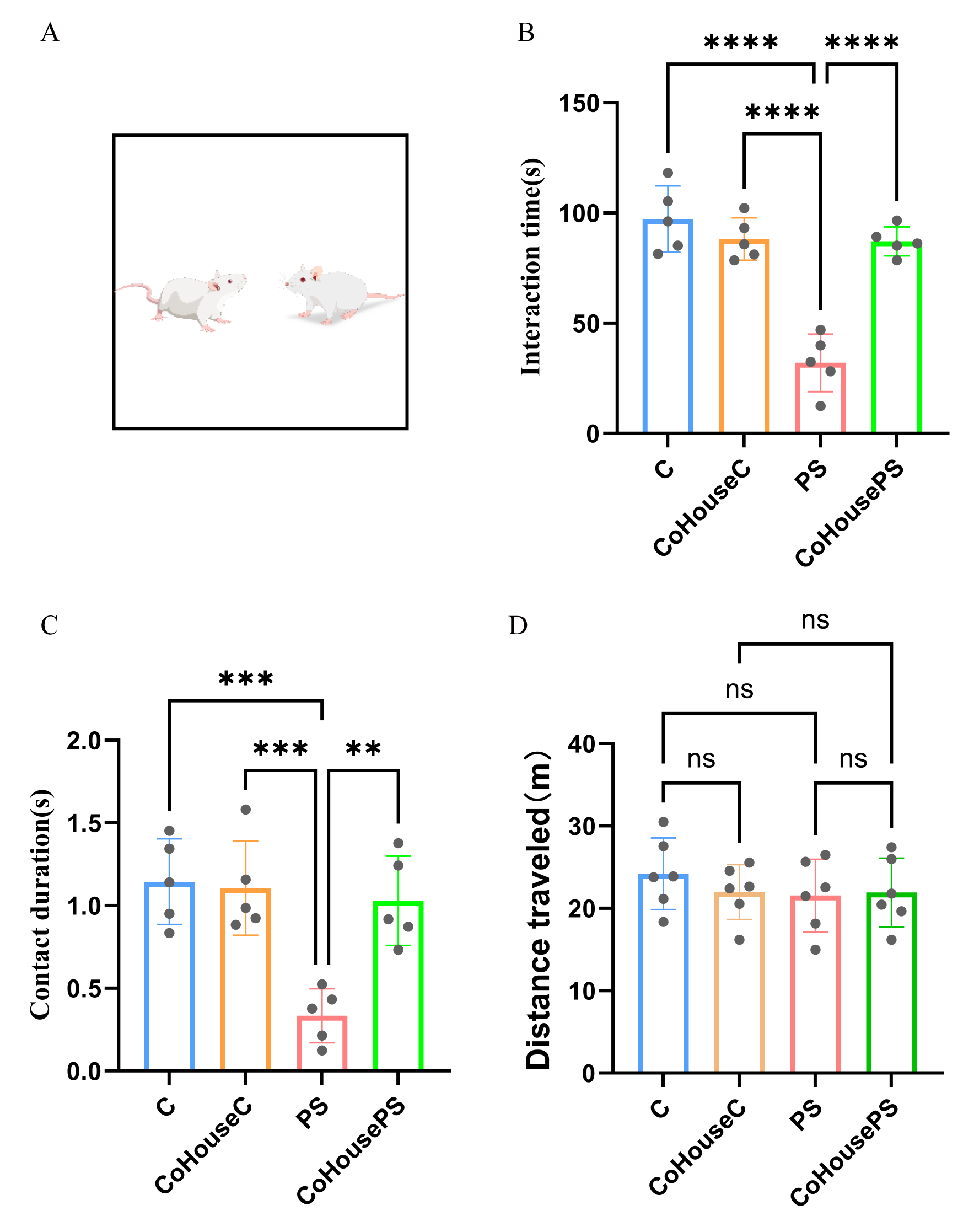


Supplementary Figure 2

A Schematic of the reciprocal social interaction task.

B-C Co-housed PS offspring showed significant improvements in reciprocal social interactions (n = 5).

D The total distance moved by rats in the empty box period of the three-box social experiment within 10 minutes (n = 6).


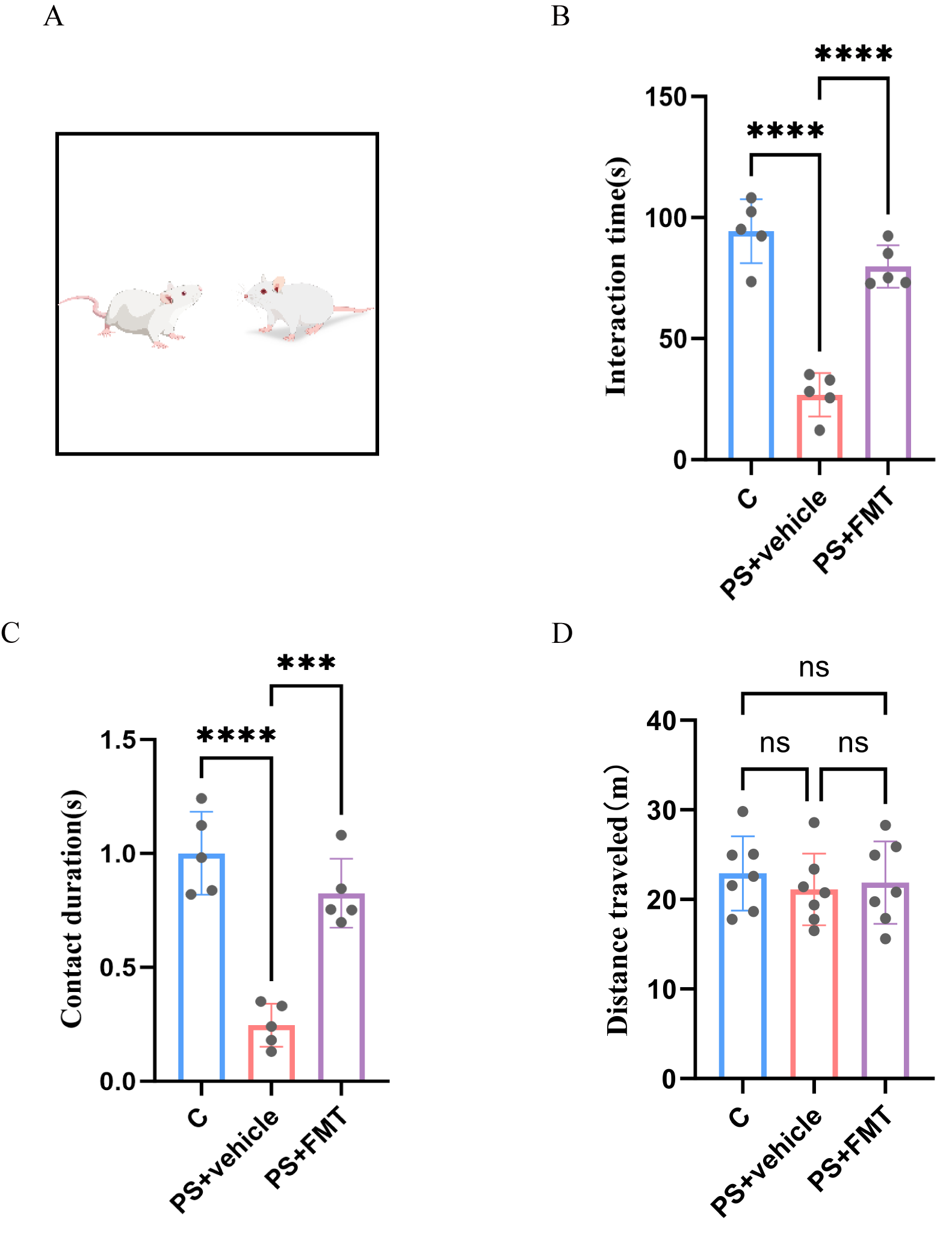


Supplementary Figure 3.

A Schematic of the reciprocal social interaction task.

B-C PS offspring receiving FMT from control donors exhibited partial restoration of reciprocal social interactions (n = 5).

D The total distance moved by rats in the empty box period of the three-box social experiment within 10 minutes (n = 7).


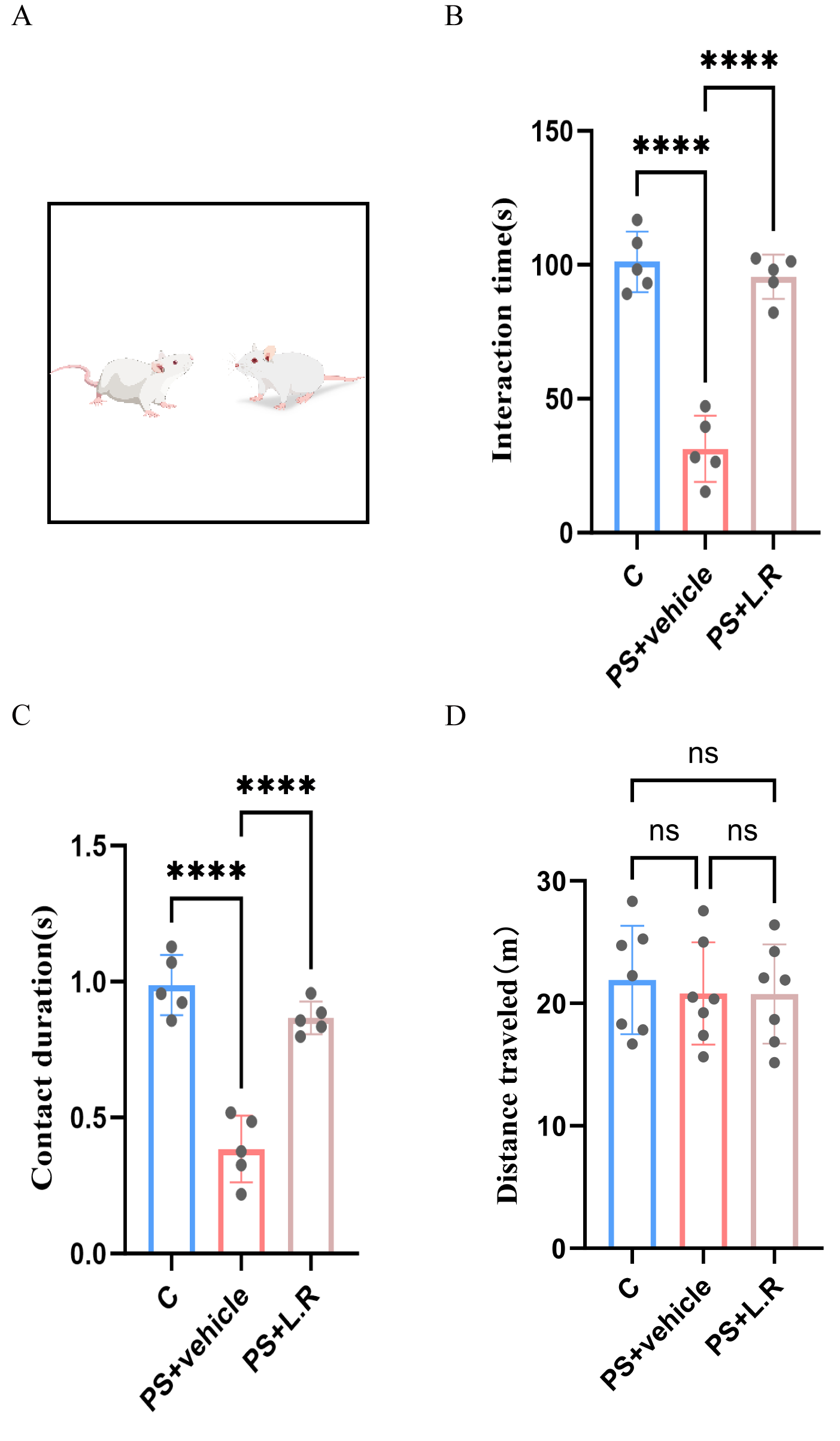


Supplementary Figure 4.

A Schematic of the reciprocal social interaction task.

B-C PS offspring supplemented with *L. reuteri* showed partial restoration of reciprocal social interactions (n = 5).

D The total distance moved by rats in the empty box period of the three-box social experiment within 10 minutes (n = 7).


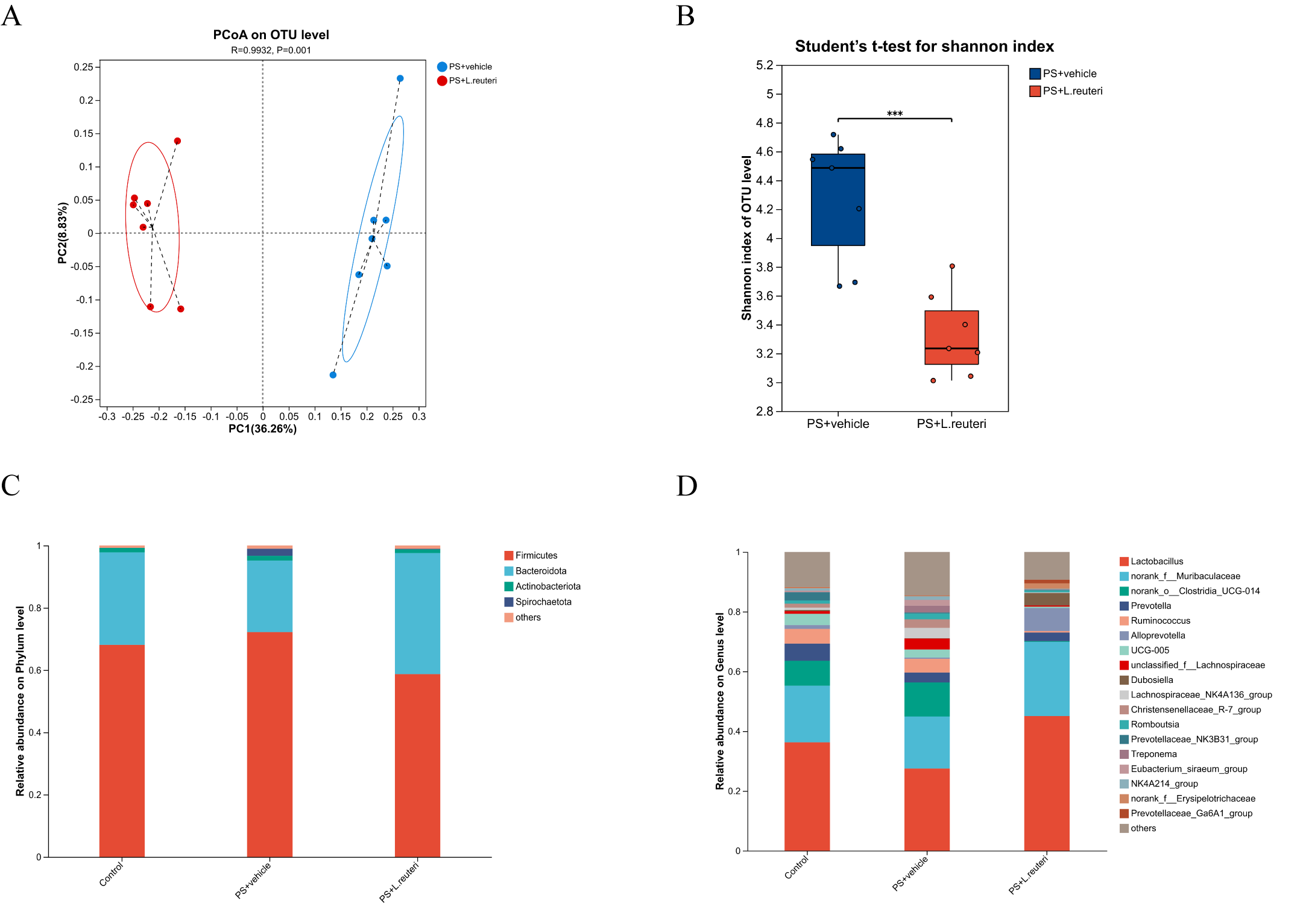


Supplementary Figure 5.

A PCoA of gut microbiota (weighted UniFrac values) revealed differential clustering between PS offspring and L. reuteri-supplemented PS offspring.

B Alpha diversity (Shannon index) showed significant differences between the PS group and L. reuteri-supplemented PS offspring.

C Relative abundance of gut microbiota at the phylum level across all groups.

D Relative abundances of gut microbiota at the genus level across all groups.


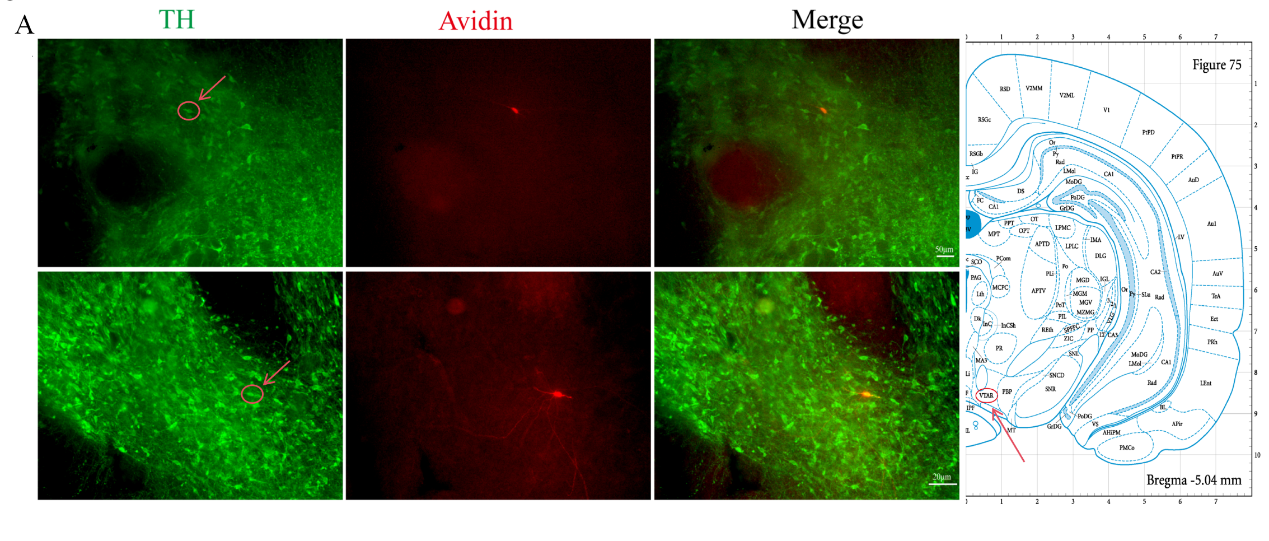


Supplementary Figure 6

A immunofluorescence staining identification of recorded dopamine neurons in the VTA.


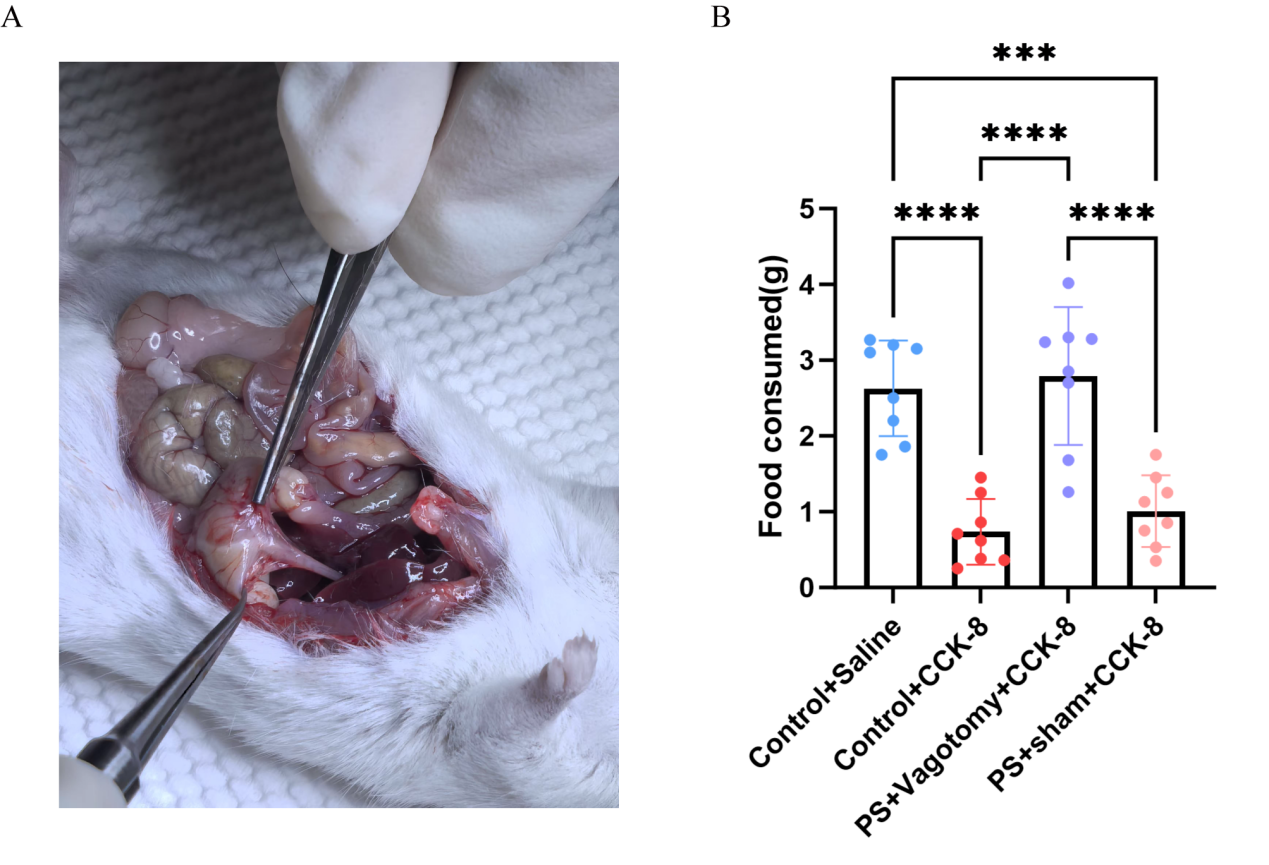


Supplementary Figure 7. Vagotomy was complete.

A Schematic illustration of subdiaphragmatic vagotomy procedure.

B Vagotomy completeness was assessed by monitoring food consumption following CCK-8 injection (n = 8).


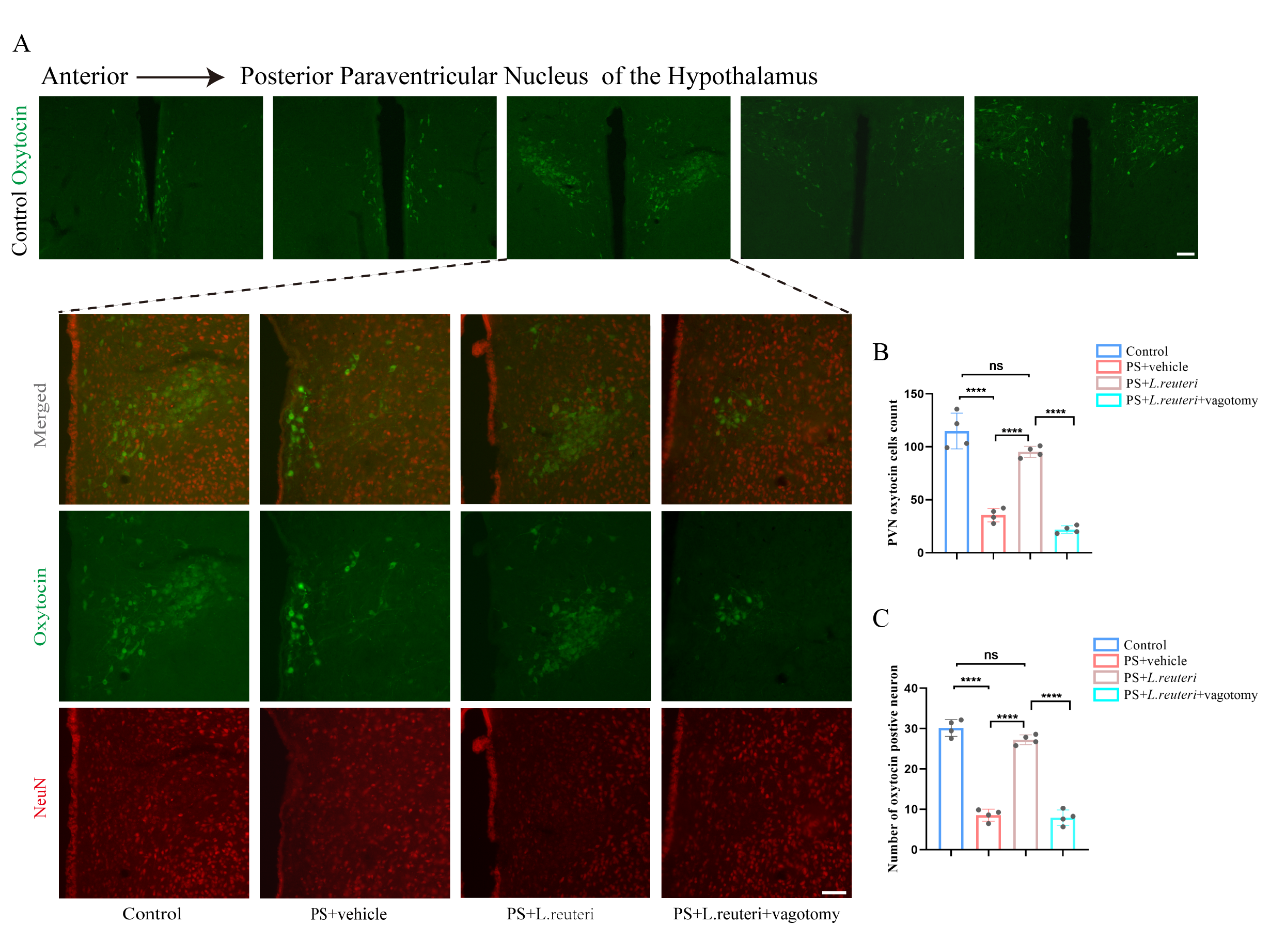


Supplementary Figure 8 Subdiaphragmatic Vagotomy Abolishes *L. reuteri*-Mediated Rescue of PVN Oxytocin Levels in PS Offspring

A Immunofluorescence co-localization of oxytocin (OXT, green) with neuronal marker NeuN (res) in PVN, demonstrating OXT's neuronal identity. B Quantification of OXT-immunoreactive cells. C Quantification of OXT+ neuron counts.
